# Correction to: Mild COVID-19 imprints a long-term inflammatory eicosanoid- and chemokine memory in monocyte-derived macrophages

**DOI:** 10.1038/s41385-022-00526-7

**Published:** 2022-05-13

**Authors:** Sina Bohnacker, Franziska Hartung, Fiona Henkel, Alessandro Quaranta, Johan Kolmert, Alina Priller, Minhaz Ud-Dean, Johanna Giglberger, Luisa M. Kugler, Lisa Pechtold, Sarah Yazici, Antonie Lechner, Johanna Erber, Ulrike Protzer, Paul Lingor, Percy Knolle, Adam M. Chaker, Carsten B. Schmidt-Weber, Craig E. Wheelock, Julia Esser-von Bieren

**Affiliations:** 1 Center of Allergy and Environment (ZAUM), Technical University of Munich and Helmholtz Center Munich, 80802 Munich, Germany; 2 Division of Physiological Chemistry 2, Department of Medical Biochemistry and Biophysics, Karolinska Institute, Stockholm, Sweden; 3 The Institute of Environmental Medicine, Karolinska Institute, Stockholm, Sweden; 4 Institute of Molecular Immunology and Experimental Oncology, University Hospital rechts der Isar, Technical University of Munich (TUM), School of Medicine, 81675 Munich, Germany; 5 Institute of Computational Biology, Helmholtz Center Munich, 85764 Neuherberg, Germany; 6 Department of Otorhinolaryngology and Head and Neck Surgery, University Hospital rechts der Isar, Technical University of Munich (TUM), School of Medicine, 81675 Munich, Germany; 7 Department of Internal Medicine II, University Hospital rechts der Isar, Technical University of Munich (TUM), School of Medicine, 81675 Munich, Germany; 8 Institute of Virology, Technical University of Munich (TUM), School of Medicine and Helmholtz Zentrum München, 81675 Munich, Germany; 9 German Center for Infection Research (DZIF), Munich partner site, Munich, Germany; 10 Department of Neurology, University Hospital rechts der Isar, Technical University Munich (TUM), School of Medicine, 81675 Munich, Germany; 11 German Center of Lung Research (DZL), Munich partner site, Munich, Germany; 12 Department of Respiratory Medicine and Allergy, Karolinska University Hospital, 141-86 Stockholm, Sweden; 13 Gunma Initiative for Advanced Research (GIAR), Gunma University, Maebashi, Japan

Correction to: *Mucosal Immunology* (2022) **15**:515–524; 10.1038/s41385-021-00482-8, published online 15 March 2022

The original version of this article contained an error in the ESM. The supplemental file titled “Supplementary information 1” is a marked version of the correct file, “Supplementary information 2”. “Supplementary information 1” was therefore removed. The authors apologize for the error. The original article has been corrected.

